# Comparison between self-reported physical activity (IPAQ-SF) and pedometer among overweight and obese women in the MyBFF@home study

**DOI:** 10.1186/s12905-018-0599-8

**Published:** 2018-07-19

**Authors:** Mohamad Hasnan Ahmad, Ruhaya Salleh, Noor Safiza Mohamad Nor, Azli Baharuddin, Wan Shakira Rodzlan Hasani, Azahadi Omar, Ahmad Taufik Jamil, Mahenderan Appukutty, Wan Abdul Manan Wan Muda, Tahir Aris

**Affiliations:** 1 Institute for Public Health, National Institutes of Health, Ministry of Health Malaysia, Kuala Lumpur, Malaysia; 2 Population Health and Preventive Medicine, Faculty of Medicine, Universiti Teknologi Mara (UiTM), Sungai Buloh Campus, Jalan Hospital, Sungai Buloh, Selangor Darul Ehsan Malaysia; 3 Faculty of Sport Science & Recreation, Universiti Teknologi Mara (UiTM), Shah Alam Campus, Shah Alam, Selangor Darul Ehsan Malaysia; 4 Center for Southeast Asian Studies, Kyoto University, Kyoto, Japan

**Keywords:** Physical activity, International physical activity questionnaire (IPAQ-SF), Pedometer, Obese women, Malaysia

## Abstract

**Background:**

Several methods have been developed to determine a person’s physical activity level. However, there is limited evidence in determining whether someone is physically active or not. This study aims to determine the level of physical activity and to compare the usage of short version International Physical Activity Questionnaire (IPAQ-SF) and pedometer among overweight and obese women who were involved in the My Body is Fit and Fabulous at home (MyBFF@home) study.

**Methods:**

Baseline and sixth month data from the MyBFF@home study were used for this purpose. A total of 169 of overweight and obese respondents answered the IPAQ-SF and were asked to use a pedometer for 7 days. Data from IPAQ-SF were categorised as inactive and active while data from pedometer were categorised as insufficiently active and sufficiently active by standard classification. Data on sociodemographic and anthropometry were also obtained. Cohen’s kappa was applied to measure the agreement of IPAQ-SF and pedometer in determining the physical activity level. Pre-post cross tabulation table was created to evaluate the changes in physical activity over 6 months.

**Results:**

From 169 available respondents, 167 (98.8%) completed the IPAQ-SF and 107 (63.3%) utilised the pedometer. A total of 102 (61.1%) respondents were categorised as active from the IPAQ-SF. Meanwhile, only 9 (8.4%) respondents were categorised as sufficiently active via pedometer. Cohen’s κ found there was a poor agreement between the two methods, κ = 0.055, *p* > 0.05. After sixth months, there was + 9.4% increment in respondents who were active when assessed by IPAQ-SF but − 1.3% reductions for respondents being sufficiently active when assessed by pedometer. McNemar’s test determined that there was no significant difference in the proportion of inactive and active respondents by IPAQ-SF or sufficiently active and insufficiently active by pedometer from the baseline and sixth month of intervention.

**Conclusion:**

The IPAQ-SF and pedometer were both able to measure physical activity. However, poor agreement between these two methods were observed among overweight and obese women.

## Background

Physical activity is defined as the body movement produced by skeletal muscles that results in energy expenditure [1]. However, the level of physical activity differs from person to person depending on his or her personal choice, which is in turn largely subject to many internal and external factors [2].

Nowadays, the prevalence of overweight and obesity is rising around the world and has become a global issue. Modernisation and advancement of transportation is believed to reduce the physical activity of people and contribute towards this epidemic [3]. The excess calories due to physical inactivity will be stored in the body as fat and if it continues, he or she will have excess body fat which will increase their body weight [4].

Based on the most recent findings from the National Health & Morbidity Survey (NHMS) 2015, more women in Malaysia were categorised as overweight and obese compared to men [5]. Excess body weight among women has always been a big concern either for their health or body image. Being physically active is highly recommended in order for women to reduce their body weight other than reducing calorie intake [6]. However, the way women are physically active might be different from men, as majority of women in Malaysia are housewives who are responsible for all household chores [7]. They may have more time, but most of their time are spent at home being sedentary or doing housework.

The assessment of physical activity is complex and troublesome to get an accurate estimation. However, the use of suitable methods in determining physical activity level is important to ensure accurate information on physical activity is obtained to produce effective intervention programmes. Several methods have been developed to determine physical activity levels. These methods can be divided into two categories. The first one is self-reported measurement such as the Global Physical Activity Questionnaire (GPAQ), the International Physical Activity Questionnaire-Short Form (IPAQ-SF) and physical activity diary. The second one is a direct measure of physical activity which includes calorimeter, accelerometer and pedometer usage [8].

However, from the available methods, there is limited evidence in determining whether someone is physically active or not, especially among women with excess body weight. This is because, previous studies have reported that there was high potential of bias on physical activity assessment especially by self-reported methods and among certain group of people [9]. Therefore, this paper aimed to determine the level of physical activity, and to compare the usage of a self-reported measure of physical activity which is the International Physical Activity Questionnaire-Short Form (IPAQ-SF) and the direct measure using pedometer among overweight and obese women living in low-cost flats.

## Methods

Baseline and sixth month data were obtained from the My Body is Fit & Fabulous at home (MyBFF@home) study. This was a community-based intervention study to combat obesity among overweight and obese women in the Klang Valley, which includes the central area of Kuala Lumpur and its adjoining cities and towns in the state of Selangor. The methodology and the details of the MyBFF@home study have been reported elsewhere [10].

### Variables used

Data on sociodemographic, anthropometry and physical activity assessment by using Short Form of International Physical Activity Questionnaire (IPAQ-SF) and pedometer were obtained. Socio demographic data included age, ethnicity, education level, marital status, number of children and household income. Meanwhile, data for anthropometry were weight, height and waist circumference. Data on weight and height were used to calculate Body Mass Index (BMI) and recoded based on World Health Organization 1998 cut-off points. Data for waist circumference were recoded according to cut-off recommendations for Asian populations [11].

Respondents were asked to complete the IPAQ-SF. The IPAQ-SF records the last 7 day recall for four intensity levels of physical activity which is vigorous-intensity activity, moderate-intensity activity, walking, and sitting. From IPAQ-SF, data were converted to Metabolic Equivalent minutes per week (MET-min/week) using the published formulation by Ainsworth et al. [12]. Compendium average MET score (Walking = 3.3 METs, Moderate Physical Activity = 4.0 METs and Vigorous Physical Activity = 8.0 METs) [13].

Respondents who met these following criteria, which is ≥3 days of vigorous activity of at least 20 min per day, or ≥ 5 days of moderate-intensity activity and walking of at least 30 min per day, or ≥ 5 days of any combination of walking, moderate-intensity and vigorous intensity activities achieving a minimum of at least 600 MET-min/week, or Vigorous-intensity activity on at least 3 days and accumulating at least 1500 MET minutes/week or ≥ 7 days of any combination of walking, moderate-intensity and vigorous intensity activities achieving a minimum of at least 3000 MET-minutes/week, were considered as active. Those respondents not meeting the above criteria were categorised as inactive [14]. Meanwhile, data on steps count from pedometer were converted into two categories of physical activity; those with less than 10,000 steps per day as insufficiently active and those with ≥10,000 steps per day as sufficiently active [15].

### Data analysis

The data was analysed using IBM SPSS version 23 for Windows (SPSS Inc., Chicago, IL, USA). Data was harmonized and recoded according to the study objective. Descriptive statistics were used for describing the characteristics of the respondent. The two by two cross-tabulation table was created and Cohen’s kappa was calculated to measure the level of agreement between IPAQ and pedometer in the classification of physical activity level using baseline data. The McNemar test was carried out to determine if there were differences on a dichotomous dependent variable which included physical activity levels by IPAQ-SF and pedometer from baseline to sixth month of the intervention.

## Results

From the 169 available respondents, 167 (98.8%) completed the IPAQ-SF and 107 (63.3%) utilised the pedometer. The average steps count from pedometer was 5196 steps per day with minimum steps recorded as only 377 and the maximum being 19,303.

By IPAQ-SF, 61.1% respondents were categorised as active. There were more physically active respondents in the 40–49 years age group, Malay ethic group, women who had secondary and high education level, women who were married, had 3–4 number of children, those with household income more than RM2,501.00, those in pre-obesity BMI category and with no abdominal obesity by waist circumference classification. About 8.4% respondent were sufficiently active from pedometer. There were more sufficiently active respondents from the younger age group (18–29 years old), Chinese ethnic group, with minimum secondary education, unmarried, with less than 2 children, income between RM1501.00 – RM2500.00, being obese II and with no abdominal obesity by waist circumference classification as shown in Table 1.

**Table 1:** Characteristics of the respondents and status of physical activity based on IPAQ-SF and pedometer, n (%)

Variable	Number of respondents	Physical activity level			
		IPAQ-SF		Pedometer	
		Inactive	Active	Insufficiently active	Sufficiently active
Total respondents	169 (100)	65 (38.9)	102 (61.1)	98 (91.6)	9 (8.4)
Age (years)					
18–29	14 (8.3)	6 (42.9)	8 (57.1)	7 (87.5)	1 (12.5)
30–39	48 (28.4)	20 (41.7)	28 (58.3)	26 (92.9)	2 (7.1)
40–49	72 (42.6)	21 (29.6)	50 (70.4)	44 (89.8)	5 (10.2)
≥ 50	35 (20.7)	18 (52.9)	16 (47.1)	21 (95.5)	1 (4.5)
Ethnicity					
Malay	143 (84.6)	54 (38.3)	87 (61.7)	90 (91.8)	8 (8.2)
Chinese	5 (3.0)	2 (40.0)	3 (60.0)	2 (66.7)	1 (33.3)
Indian	18 (10.7)	7 (38.9)	11 (61.1)	4 (100.0)	0 (0.0)
Others	3 (1.8)	2 (66.7)	1 (33.3)	2 (100.0)	0 (0.0)
Education					
Primary and lower	29 (17.3)	16 (55.2)	13 (44.8)	16 (94.1)	1 (5.9)
Secondary and higher	139 (82.7)	49 (35.8)	88 (64.2)	82 (91.1)	8 (8.9)
Marital status					
Married	153 (90.5)	57 (37.5)	95 (62.5)	88 (92.6)	7 (7.4)
Not married/ widowed/separated	16 (9.5)	8 (53.3)	7 (46.7)	10 (83.3)	2 (16.7)
Number of children					
≤ 2	38 (22.6)	17 (44.7)	21 (55.3)	18 (85.7)	3 (14.3)
3–4	82 (48.8)	26 (32.1)	55 (67.9)	47 (90.4)	5 (9.6)
≥ 5	48 (28.6)	21 (44.7)	26 (55.7)	33 (97.1)	1 (2.9)
Household income					
≤ RM 1500.00	80 (48.2)	38 (48.7)	40 (51.3)	43 (95.6)	2 (4.4)
RM 1501.00–2500.00	54 (32.5)	19 (35.2)	35 (64.8)	34 (87.2)	5 (12.8)
≥ RM 2501.00	32 (18.3)	7 (21.9)	25 (78.1)	20 (90.1)	2 (9.9)
Obesity status					
Pre-obesity	73 (43.2)	27 (37.5)	45 (62.5)	40 (93.2)	3 (6.8)
Obese I	58 (34.3)	22 (38.6)	35 (61.4)	33 (91.7)	3 (8.3)
Obese II	38 (22.5)	16 (42.1)	22 (57.9)	27 (88.9)	9 (11.1)
Waist circumference					
≤ 80 cm	12 (7.1)	2 (16.7)	10 (83.3)	7 (87.5)	1 (12.5)
> 80 cm	157 (92.9)	63 (40.6)	92 (59.4)	91 (91.9)	8 (8.1)

Cohen’s κ was used to determine if there was an agreement between the two methods in determining physical activity levels among the respondents. There was a poor agreement between these two methods, Cohen's κ = 0.055, *p* > 0.05, as shown in Table 2.

**Table 2:** Measure of agreement between IPAQ-SF and pedometer on physical activity classification n (%)

		Pedometer		Cohen’s κ	*p*-value
		Insufficiently active	Sufficiently active		
IPAQ-SF	Inactive	42 (95.5)	2 (4.5)	0.055	0.229
	Active	56 (88.9)	7 (11.1)		

*p* < 0.05, significant for Cohen's kappa test

Over the course of sixth months, there was some increase in the number respondents who were active when assessed by IPAQ-SF but a slight reduction for respondents being sufficiently active when assessed by pedometer. The exact McNemar’s test showed that there was no significant difference in the proportion of respondents inactive and active by IPAQ-SF or sufficiently active and insufficiently active by pedometer from the baseline and sixth month of intervention as shown in Table 3.

**Table 3:** Change in physical activity level by IPAQ-SF and pedometer from baseline to sixth month n (%)

Test	Category	Intervention period		Percent changes	McNemar *p*-value
		Baseline	Sixth month		
IPAQ-SF	Inactive	65 (38.9)	36 (29.5)	−9.4	0.067
	Active	102 (61.1)	86 (70.5)	+ 9.4	
Pedometer	Insufficient active	98 (91.6)	39 (92.9)	+ 1.3	0.999
	Sufficient active	9 (8.4)	3 (7.1)	−1.3	

*p* < 0.05, significant for McNemar test

## Discussion

This recent study reported that levels of physical activity among overweight and obese women who live in low-cost flats in Klang Valley was higher when measured using the self-reported measure (IPAQ-SF) compared to direct measurement by pedometer. Previous study comparing subjective (IPAQ) and objective (accelerometer) measure of physical activity also found almost similar finding, whereby IPAQ is more likely to overestimate actual physical activity by its limited ability to classify adults into low and high categories of physical [14]. Over the 6 months intervention in MyBFF@home program, the level of high physical activity assessed by pedometer saw a slightly drop compared to the IPAQ-SF. This is because the type of intervention in MyBFF@home mainly focuses on 12 step exercise using pillow dumbbell exercise, which activity is not capture by pedometer [10].

The IPAQ-SF development was premised on the need to develop international population measurement to assess ‘total physical activity’ and categorize respondents into various levels of physical activity [15]. It relies on ability to recall and honesty of the respondents to report the physical activity which they have engaged in over the past 7 days [16]. A previous study also reported that the IPAQ-SF does not appear to be a good indicator of individual physical activity behaviour especially among older adults but is better suited for larger population-based samples [17].

The pedometer on the other hand, does not rely on self-recall of physical activity or subjective assessment of exercise intensity, in contrast to physical activity questionnaires [18]. This device directly senses the body motion and counts the footsteps [19]. Many previous studies reported that pedometers are a good proxy to physical activity because it reduces the subjectivity inherent in survey methods. It can be applied to large groups of individuals and also allows results from research studies to be readily translated into physical activity levels [18, 20]. However, the limitation of pedometers is that it does not measure all types of physical activity, and it is acknowledged that they do not capture swimming, cycling, and weight lifting [21]. Studies also found inverse trends in physical activity among obesity status. Those who are pre-obese were found to be more active when assessed by self-reported measure but insufficiently active when assessed by direct measure [22, 23]. Conversely, those who were obese type II were found to be more inactive when assessed by IPAQ-SF but sufficiently active when assessed by pedometer.

The findings coincide with a systematic review conducted in 2008 which reported correlations between self-reported and direct measures were generally uncertain and it can be negatively or positively correlated, ranging from − 0.71 to 0.96. The same systematic review also concluded that the self-reported measures of physical activity were both higher and lower than directly measured levels of physical activity, which poses a problem for both reliance on self-reported measures and for attempts to correct for self-reported – directly measured differences [8].

There is also a systematic review on validity of IPAQ-SF reporting that the correlation between the IPAQ-SF and objective measures of activity or fitness was lower than the acceptable standard in the large majority of studies. Furthermore, the IPAQ-SF typically overestimated physical activity as measured by objective criterion by an average of 84%. Hence, the evidence to support the use of the IPAQ-SF as an indicator of relative or absolute physical activity is weak [24].

A critical appraisal paper on assessment of physical activity published in 2009 reported that doubly labelled water method was the gold standard in assessing physical activity. However, this method gives high interference to the respondent, is very expensive and the data does not provide specific information on daily physical activity. Questionnaires which use self-reported methods show a low reliability and validity but can be adequately applied as an activity-ranking instrument [25]. Pedometer or accelerometer was also a good instrument for assessing physical activity but it needs to be upgraded so that it can provide not just step count data, but also information on body posture and activity recognition to allow objective assessment of subjects’ habitual activities, options for a healthy change, and effects of the follow-up of any changes [25].

There are several strengths and limitations in this study. The response rate for IPAQ-SF was good but the response rate for pedometer was low. However, it is still adequate to perform statistical analysis at the baseline. The level of motivation and awareness among the participants could be one of the barriers to using the pedometer every day. Almost half of the respondents have more than five children and they spent most of their time doing housework at home or attending to their children. The other limitation is the distribution of the respondents is not equal across sociodemographic variables. This imbalance does not allow reliable inferential statistical tests to be applied to the data especially for the sixth month. Thus, the results should be interpreted with caution.

## Conclusion

In summary, the physical activity status among overweight and obese women was high using the self-report method, IPAQ-SF as compared to the direct method using pedometer. Overall, we found that physical activity levels among overweight and obese women who are living in low-cost flats in Klang Valley to be low. It was also found that there is a lack of agreement in determining physical activity level by IPAQ-SF and pedometer. Therefore, the usage of the IPAQ-SF or pedometer in measuring physical activity must be focused on the objective since both methods measure different sides of physical activity.

## Abbreviations

GPAQ: Global Physical Activity Questionnaire
IPAQ-SF: International Physical Activity Questionnaire –Short Form
MyBFF@home: My Body is Fit and Fabulous at Home
NHMS: National Health and Morbidity Survey
